# Enhancing healthcare accessibility through telehealth for justice impacted individuals

**DOI:** 10.3389/fpubh.2024.1401950

**Published:** 2024-08-08

**Authors:** Karmen S. Williams, Marianna J. Singh, Johanna E. Elumn, Megan Threats, Yongjie Sha, Terika McCall, Karen Wang, Bria Massey, Mary L. Peng, Kevin Wiley

**Affiliations:** ^1^Department of Health Policy and Management, Graduate School of Public Health and Health Policy, City University of New York, New York, NY, United States; ^2^SEICHE Center for Health and Justice, General Internal Medicine, Yale School of Medicine, New Haven, CT, United States; ^3^School of Information, University of Michigan Ann Arbor, Ann Arbor, MI, United States; ^4^Division of Health Informatics, Department of Biostatistics, Yale School of Public Health, New Haven, CT, United States; ^5^Center for Population Health IT, Department of Biomedical Informatics and Data Science, Johns Hopkins School of Medicine, Baltimore, MD, United States; ^6^Department of Global Health and Social Medicine, Harvard Medical School, Boston, MA, United States; ^7^Department of Healthcare Leadership and Management, College of Health Professions, Medical University of South Carolina, Charleston, SC, United States

**Keywords:** telehealth, accessibility, justice impacted individuals, prison health, Medicaid, digital health technology

## Abstract

Telehealth is a great tool that makes accessing healthcare easier for those incarcerated and can help with reentry into the the community. Justice impacted individuals face many hardships including adverse health outcomes which can be mitigated through access to telehealth services and providers. During the federally recognized COVID-19 pandemic the need for accessible healthcare was exacerbated and telehealth use surged. While access to telehealth should be considered a necessity, there are many challenges and barriers for justice impacted individuals to be able to utilize this service. This perspective examines aspects of accessibility, pandemic, policy, digital tools, and ethical and social considerations of telehealth in correctional facilities. Carceral facilities should continue to innovate and invest in telehealth to revolutionize healthcare delivery, and improve health outcomes for justice impacted individuals.

## 1 Introduction

Healthcare in prisons is a complex issue influenced by various factors such as overcrowding, limited resources, and the unique healthcare needs of justice impacted individuals. Overcrowding in poor living conditions and limited access to healthcare has led to an increase in the spread of diseases in carceral facilities. Although the terms jails and prisons are often used interchangeably, jails traditionally have shorter-term stays but with similar exposures to the healthcare needs of prisons. Additionally, mental health is exacerbated by isolation, trauma, and lack of access (1–3). A study conducted in 2009 in the US compared the health outcomes of justice impacted individuals to non-justice impacted individuals and found that the justice impacted individuals had significantly higher rates of non-communicable diseases such as hypertension, asthma, arthritis and cervical cancer (4). A systematic review indicated nearly 40% of justice impacted individuals had chronic medical conditions, and of those 40%, nearly 14% had not received any form of medical evaluation or care while in custody (5).

Access to healthcare services in prisons varies widely depending on factors such as jurisdiction, budget constraints, and staffing levels (1, 2). While some prison systems provide comprehensive healthcare services, others struggle to meet the basic medical needs of inmates (6). Other factors include limited patient-centered approaches due to security restrictions, social and cultural factors such as stigma, and costs associated with transportation and limiting potential escapes (4). A report by the Office of General Inspections indicated nearly 20% of correctional facility health positions are left vacant limiting the number of patients that can be treated (7). With this deficit many facilities look to outside clinics and organizations for help but getting patients seen at these facilities takes a long time. One facility reported an average wait time of 114 days before patients can be seen (7).

Telehealth allows clinical and non-clinical health providers to visit with a person without physically being in an office (8). Although telemedicine is often used interchangeably with telehealth, telemedicine refers to telecommunication technologies used for clinical care activities, such as diagnosis and medical treatment related activities (8). This type of care is primarily completed online with internet access to computers, tablets, or smartphones. Telehealth visits range from lab and x-ray results, mental health, skin conditions, prescription management, to post-surgical follow-ups and physical therapy (9). Although COVID-19 telehealth use increased by 766% in the first 3 months of the pandemic, telehealth is not a new technology. Telehealth services can be traced back to the 1970s in the United States, but was limited due to cost (10, 11). However, during this time, the expansion of services progressively extended to prisons, and increased in offerings. Such as mentoring intensive care, telepsychiatry and teleconsultations between hospitals and prisons (12). In the 1990s, the United States Department of Justice Office of Justice Programs released a tool guide for correctional facilities to use to implement telehealth services. The Department of Justice cited and recognized that “telemedicine could play an important role in delivering quality health care in correctional systems” (11, 13).

The benefits of telehealth for justice impacted individuals include reduction in overall wait time for medical care referrals, increase in access to outpatient visits, such as psychiatry services, eliminates the need to transport to medical appointments, lowers costs, and assist with care management, which impacts morbidity and mortality (13). Additionally, telehealth can help those on the pathway of reentry as it builds trust with community-based providers and provides a familiar point of contact when leaving a correctional facility (14). Telehealth also showed benefits for correctional facilities by reducing costs for transportation, security, and personnel (11).

Although telehealth can benefit communities, including correctional facilities, there are increased barriers to access health care, even with the uptake of telehealth. These same barriers are exacerbated by ethical and social considerations for justice impacted individuals. Digital health technology and tools can assist in reducing these barriers, but further interventions and considerations are necessary.

The aims of this perspective are to characterize the access to telehealth services in correctional facilities, and to discuss the benefits and considerations for telehealth access to improve care for justice impacted individuals. The references identified in this perspective piece are not meant to be exhaustive but specifically selected to characterize the topics related to telehealth use and access in correctional facilities.

## 2 Telehealth accessibility for justice impacted individuals

### 2.1 Technology and internet access

For most Americans, technology has become ubiquitous with basic daily function. While the COVID-19 pandemic exposed the severity of the digital divide, it also revealed a great potential for technology to bridge those inequalities. Rising concerns around restricted technology access during incarceration were especially notable (15, 16). From mediating access to educational resources, connecting with distant family, accessing healthcare, and developing digital literacy skills, the benefits of technology access in prisons and jails are manifold (17, 18).

**Figure 1:**
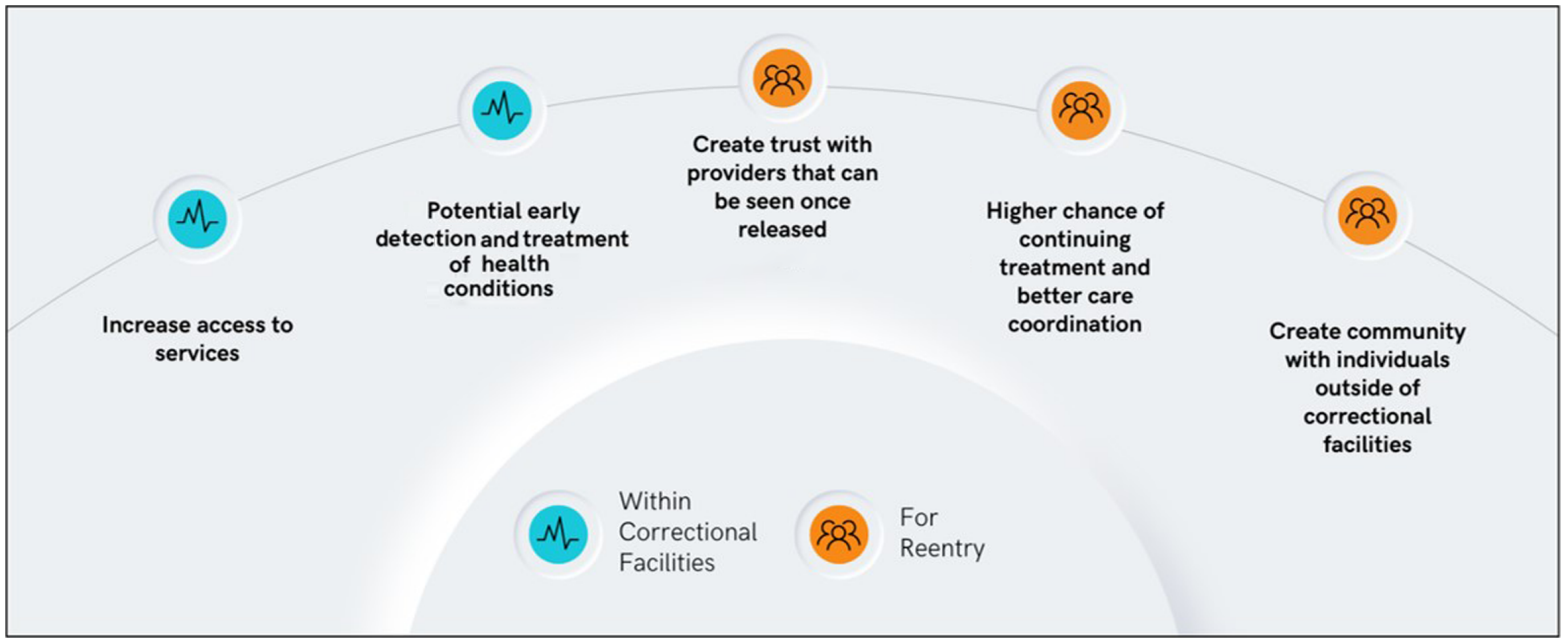
Benefits of telehealth accessibility within correctional facilities and for reenty preparation.

The intense social isolation at the peak of the pandemic was assuaged by virtual family visits, providing essential connections to loved ones (19). Telehealth was also utilized for medical care without sending the person outside of the facility. Telehealth has increased access to care, including behavioral health services and specialized treatment typically unavailable within carceral facilities (20–22). Future expansion of telehealth could allow justice impacted individuals to further engage in behavioral health services with their families (22, 23). This use of digital technology during the pandemic provides clear evidence of how technology might be used moving forward to provide needed services and increase the chances of successful reentry (18, 24, 25). Figure 1 illustrates the benefits of telehealth access within carceral systems and for reentry.

Despite the existing evidence, technology access continues to be heavily contested. Restricted Wi-Fi access on designated devices is available in some facilities, but many simply deny internet access (26). Some states have policies related to use of computers, internet access, and social media, but there are few standardized regulations regarding digitization in carceral environments (27). In cases of the former, the use of tablets designed by for-profit companies is stifled by paywalls (16). Some facilities have continued to use virtual visits in addition to in-person visits (28).

Amidst all this complexity, there must also be concern that even after release, technology use may be stifled under the surveillance of community carceral providers—halfway houses, probation, and parole officers—and by lack of internet access, smart devices, and digital literacy (26, 29, 30). Without access to digital technology during incarceration, people leave carceral facilities unprepared for life in a world where most interactions, from finding a job or a home to connecting with service providers and family, require access to digital devices and the internet (18).

### 2.2 COVID-19 pandemic

The COVID-19 pandemic had a profound impact on the health of justice impacted populations. Justice impacted populations experienced a disproportionate burden of COVID-19 in comparison to the general U.S. population. By June 2020, the COVID-19 infection case rate was 5.5 times higher for justice impacted individuals in prisons than the U.S. general population case rate of 587 per 100,000 (31). The disproportionate impact has been attributed to close habitation, the inability to effectively physically distance, overcrowding, poor ventilation, limited access to hygiene products, frequent transfers of incarcerated individuals between facilities, and the migration patterns of correctional staff (32). In efforts to reduce the rapid transmission of COVID-19 among justice impacted populations, correctional facilities limited transport for non-life threatening healthcare related visits and activities (33).

In response to these challenges, the rapid proliferation of telehealth in correctional facilities, due to physical distancing policies, Medicare expansion for telehealth services, and some funding specifically for this effort, emerged as a promising innovation to improve access to care for justice impacted populations to meet medical and behavioral health needs (34). Telehealth interventions utilized during the COVID-19 pandemic included the use of information and communication technologies, such as video conferencing technology, telephone calls, and tablets, to conduct consultations, medication management, evaluations, and therapy (35–38). Several facilities utilized telehealth to deliver mental healthcare services, to facilitate the treatment of substance use disorders, and to improve access to routine healthcare for individuals incarcerated in rural areas (20, 22, 23, 37, 39, 40). Since the progression of the COVID-19 pandemic, key legislation in several states, such as Michigan, Minnesota, and North Carolina, has been introduced to expand access to telehealth in correctional facilities and to improve healthcare outcomes for justice impacted individuals (41, 42).

### 2.3 Medicaid policies

Few, if any, studies have examined the effect of Medicaid policy on the availability and accessibility of telehealth in carceral systems. However, these policies may have an outsized impact on patient care quality and health outcomes. While Medicaid is not a significant funding source for healthcare in jails and prisons, its imprimatur as a large insurer could improve access to care for many underinsured and underresourced patient populations (43).

In some cases, Medicaid prohibits the use of funds for justice impacted individuals in a public institution except when the individual is a “patient in a medical institution” organized to provide medical care (27). States have the latitude to submit customized Medicaid program modifications adopted and operationalized once approved by the Centers for Medicare and Medicaid Services.

These modifications are waivers under Section 1115 of the Social Security Act (44). Commonly known as 1,115 waivers, these demonstration projects may facilitate or restrict Medicaid benefits necessary to access needed care, depending on the state (45, 46). For example, Indiana’s 2015 1,115 Medicaid waiver expedited coverage for justice impacted adults by initiating Medicaid applications while in custody and temporarily suspending coverage where it was previously just discontinued (47). Researchers (48) found that this waiver was associated with increased coverage for justice impacted adults. Notably, Indiana’s Medicaid program levies penalties on beneficiaries who lapse on their coverage (48).

Research evaluating the impact of 1,115 waiver programs and subsequent demonstration projects on healthcare outcomes is still developing, and much of it excludes telehealth in jails or prisons. Thus, there are few incentives given 51 different approaches to Medicaid, i.e., reimbursement opportunities for telehealth providers who serve justice impacted populations.

### 2.4 Digital technology designs

Telehealth has been used to support justice impacted individuals while in prison and after their release by facilitating patient-provider communication and shared decision making (e.g., during telemedicine visit) (49). More recently, greater attention has been focused on the use of digital health technology to support management of health conditions (e.g., smartphone app to manage cardiovascular disease) (50), and facilitate connections to community resources and peer support (e.g., smartphone app to support individuals during their reentry period and transition back to the community) (51, 52). Digital health tools and services can benefit individuals with a history of incarceration, however, people with this lived experience must be included in the design process to enhance usefulness, implementation success, and sustainability.

Studies have demonstrated needed design enhancements to improve use of digital health technologies for justice impacted individuals (53, 54). User-centered design (UCD) methods can be used to create telehealth tools that are context-specific, culturally sensitive, effective and engaging. UCD is an iterative design process, and the users’ and their needs are the focus in each step of the process, which includes understanding the context of use, specifying the user requirements, creating design solutions, and evaluating the telehealth tool (55). Failure to include intended users in the design process may result in the development of a tool that not only fails to meet the intended users’ needs but may not be context or culturally-relevant or trauma-informed, and may maintain the status quo and/or contribute to intervention-generated inequalities (56). A current study codesigning a mobile app with individuals with a history of incarceration to provide support as they rejoin their communities found there was need for content and features that allow users to easily access resources for employment, housing, healthcare, and medical needs (including mental health and substance use), community health workers, formal and informal support, and easy navigation of Department of Corrections Rights (57).

There are many considerations for implementation of telehealth tools to support justice impacted individuals. Access to internet enabled devices (e.g., smartphone), broadband internet access, preferred modes of communication, privacy and confidentiality concerns, and the digital and health literacy of the intended users must be considered (53). One caveat is that justice impacted individuals may have infrequent access to devices to use telehealth services and resources. This may be due to monitoring by individuals in restrictive community settings (e.g., halfway house) that limit access to personal devices, such as smartphones. Therefore, options to save data (e.g., patient reported outcomes) to cloud storage, and additional security (e.g., require personal identification number to unlock app after a specific time of inactivity has passed) should be considered when developing telehealth tools to support justice impacted individuals.

### 2.5 Ethical, legal, and social considerations

Introducing telehealth communication in correctional facilities offers significant benefits for enhancing healthcare outcomes for justice impacted individuals (13). However, this endeavor also raises ethical, legal, and social concerns that necessitate thorough consideration to ensure a lasting and sustainable impact. Limited research articulates the extent of the risk these implications pose for justice impacted individuals. However, there are some researchers who have voiced opinions on the advantages and disadvantages of technology in carceral environments (58). Health researchers, policymakers, and correctional administration must also consider the ethical, environmental, technological, and operational concerns when introducing and implementing digital health technologies, like telehealth services, for justice impacted individuals to maintain equitable and fair practices and standards.

#### 2.5.1 Environmental challenges

Ensuring the security and privacy of telehealth services necessitates providing sufficient private space for telehealth visits (59). Inadequate space not only raises substantial privacy and security concerns but also discourages patients with specific conditions or diseases, such as HIV/AIDS, behavioral health issues, and contraception needs, from sharing sensitive health information remotely (59). As a result, justice impacted individuals with specific health conditions may be at a greater safety risk. Inadequate privacy measures during telehealth visits could lead to breaches of sensitive health information, further exacerbating their vulnerability within the correctional facility.

#### 2.5.2 Technology issues

Acknowledging and understanding that telehealth services are inherently vulnerable to data security issues such as hacking of video visits are crucial (59). These risks are further amplified in correctional facilities where adequate security measures for telecommunication devices may not be in place. Justice impacted individuals are particularly susceptible to such data breaches due to the absence of encrypted telecommunication tools and features. Although, there are telecommunication companies that have distributed free tablets to justice impacted individuals in various states, there has also been a history of experiencing data breaches among these same telecommunication companies (58). These breaches allow hackers to access sensitive information and record private conversations between individuals and their attorneys, thereby violating client-attorney privilege (58). Ensuring that justice impacted individuals can access free tablets to communicate electronically without fear of self-incrimination is paramount.

Moreover, utilizing justice impacted individuals’ data for business or marketing purposes, enabling correctional facilities to distribute, transfer, or even sell content and related information to other parties is a major concern (58). Prevention or prohibition of such practices is important because of the compromise to the privacy and dignity of justice impacted individuals, potentially subjecting them to exploitation and further marginalization within the criminal justice system. Further, protections and education about using technologies in correctional facilities is a necessity.

#### 2.5.3 Operational concerns

Access to telecommunication technology and digital health literacy is vital to ensuring equitable and optimal healthcare outcomes. Health literacy is increasingly acknowledged as crucial in managing chronic diseases and utilizing healthcare services (60). However, justice impacted individuals often have lower health literacy rates. Health-related materials should be easy to understand, as comprehension of the information may help individuals to be better informed and improve management of health conditions (60).

Furthermore, it is essential to ensure that justice impacted individuals have access to specialized training on telehealth privacy and security. Telehealth equipment, software, and devices are integral to the organization’s security management plan, necessitating annual security risk assessments (59).

## 3 Discussion

Overcrowding, limited resources, and unique health care needs are just a few of the issues related to health in prisons. Since the 1990s, the DOJ recognized the vital role that telehealth has in delivering quality health care in correctional facilities (14). This perspective focused on characterizing telehealth service access in correctional facilities, and the benefits and considerations for telehealth for justice involved individuals.

**Figure 2:**
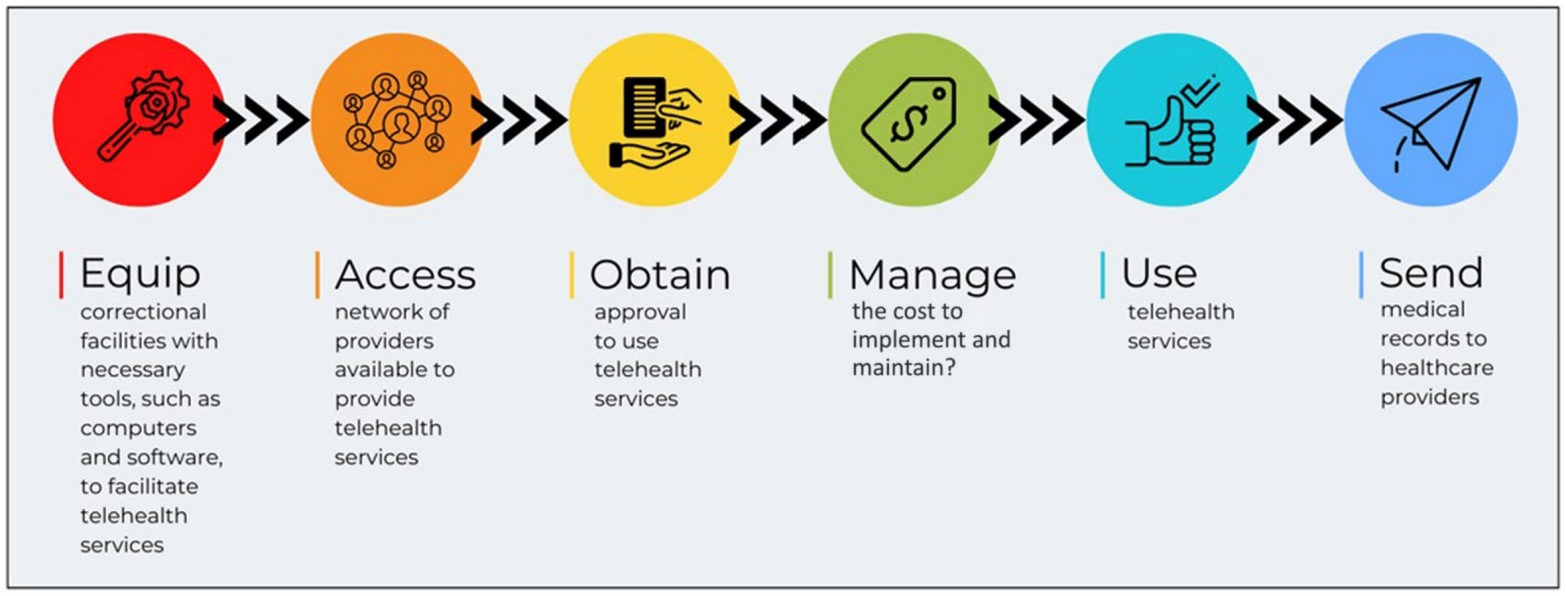
Proposed telehealth accessibility workflow for justice impacted individuals.

As depicted in Figure 2, a recommended telehealth accessibility workflow for justice impacted individuals is pertinent for improved care through the telehealth use. Correctional facilities should be equipped with the proper tools to provide telehealth services, improve access to providers through better networks, obtain approvals and set regulations that allow for reentry readiness, manage costs through commissary funds and time allotments, use the telehealth services to the advantage of both the facility and justice impacted individual, and share medical records with healthcare providers for timely coordination of care.

Recommendations for improving care through telehealth services in correctional facilities:

- With rehabilitation being a large part of the mission of correctional facilities, a special consideration for maintaining connection to families and health care providers is important and can be achieved through access to digital technology tools. Without access to digital technology during incarceration, the risk of being unprepared for life in a world where most interactions, from finding a job to a home to connecting with service providers and family, require access to digital devices and the internet (18). Correctional facilities in each state must revise regulations on digital technologies and tools in correctional facilities to better prepare justice involved individuals for reentry into their communities.
- The exacerbation of already inadequate conditions of overcrowding and staffing inability, telehealth can assist with reducing the burdens of care on staff, especially during times of emergencies, such as during the COVID-19 pandemic. Correctional facilities should invest time into emergency readiness planning that can also be effective in regular operations.
- The impact of regulations, such as 1,115 waiver programs have proven to exclude telehealth in jails and prisons and provide few incentives for reimbursement for telehealth providers who provide care for justice impacted populations. Extending telehealth care in carceral settings with support of legislation and reimbursement will improve the care for justice impacted individuals.
- Although there is no ‘one-size-fits-all’ solution for policies or implementation of technology in correctional facilities, scalable technology solutions, like telehealth, can be beneficial and relatively simple for all carceral facilities. Investing in relevant technologies that can be scalable and customizable is pertinent to improving continuity of care.
- Inclusion of justice impacted individuals in the digital tool design and implementation process will reduce unintended inequalities, such as inadequately providing contextual or culturally-relevant or trauma-informed interventions that create environments of patient care (56).
- The COVID-19 pandemic highlighted that overcrowding in correctional facilities exposes justice impacted individuals to infectious diseases due to cramped living conditions and inadequate protective measures (61). Public health officials emphasize that reducing incarceration rates is the most effective approach to mitigate these risks and create safer living conditions for justice impacted individuals (62). A multi-faced approach such as, prioritizing both de-incarceration and telehealth services together will have a higher likelihood of better health outcomes.
- Although the ongoing discussion of privacy and data ownership is ubiquitous, the conversation should extend to justice impacted individuals having access and control over their data, alternative access than from paid sponsorships that seek to use the data and ensure digital technologies are equipped with security measures and encryption methods. Correctional facilities should also ensure that documentation for telehealth services is standardized and meets recommended requirements (59). Both parties must be aware and willing to ensure the risks are mitigated, and justice impacted individuals’ data, identity, and privacy are protected. Correctional facilities must be vigilant during the implementation and maintenance of health technologies to reduce privacy and scams on the justice involved populations.

Telehealth accessibility can improve the care of justice impacted individuals both in correctional facilities and for reentry. This includes equipping correctional facilities with the proper tools that are designed with the user, providing accessibility with security and privacy, obtain approval for telehealth services, managing costs, using the telehealth services, and collaborating with medical providers to share medical records for care coordination.

## 4 Conclusion

Optimizing the use of telehealth in prisons requires a multifaceted approach that addresses accessibility, regulatory, technological, social and ethical, and collaboration challenges. By strengthening technological infrastructure, providing adequate access to digital tools, addressing the unique public health concerns in correctional facilities, identifying health insurance concerns, tailoring telehealth platforms to justice impacted individuals in correctional settings, providing training and support, and ensuring regulatory compliance and security, correctional facilities can enhance the delivery of healthcare services to justice impacted individuals. With continued innovation and investment, telehealth has the potential to revolutionize healthcare delivery in carceral facilities, improving health outcomes and promoting positive rehabilitation for justice impacted individuals.

## Data availability statement

The original contributions presented in the study are included in the article/supplementary material, further inquiries can be directed to the corresponding author.

## Author contributions

KWs: Writing – original draft, Writing – review & editing. MS: Writing – original draft, Writing – review & editing. JE: Writing – original draft, Writing – review & editing. MT: Writing – original draft, Writing – review & editing. YS: Writing – original draft, Writing – review & editing. KWa: Writing – original draft, Writing – review & editing. TM: Writing – original draft, Writing – review & editing. BM: Writing – original draft, Writing – review & editing. MP: Visualizations, Writing – review & editing. KWi: Writing – original draft, Writing – review & editing.
